# Motorcycle helmet effectiveness in reducing head, face and brain injuries by state and helmet law

**DOI:** 10.1186/s40621-016-0072-9

**Published:** 2016-03-07

**Authors:** Cody S. Olsen, Andrea M. Thomas, Michael Singleton, Anna M. Gaichas, Tracy J. Smith, Gary A. Smith, Justin Peng, Michael J. Bauer, Ming Qu, Denise Yeager, Timothy Kerns, Cynthia Burch, Lawrence J. Cook

**Affiliations:** 1 Department of Pediatrics, Division of Critical Care, University of Utah School of Medicine, 295 Chipeta Way, Salt Lake City, UT 84108 USA; 2 Department of Biostatistics and Kentucky Injury Prevention and Research Center, University of Kentucky College of Public Health, 333 Waller Ave., Suite 206, Lexington, KY 40504 USA; 3 Minnesota Department of Health, 85 East Seventh Place, Suite 220, St. Paul, MN 55164 USA; 4 Health and Demographics Section, South Carolina Revenue and Fiscal Affairs Office, Rembert C. Dennis Building, 1000 Assembly Street, Suite 240, Columbia, SC 29201 USA; 5 Center for Injury Research and Policy, The Research Institute at Nationwide Children’s Hospital, The Ohio State University College of Medicine, 700 Children’s Drive, Columbus, OH 43205 USA; 6 Connecticut Department of Public Health, Community, Family and Health Equity Section, Public Health Initiatives Branch, 410 Capitol Avenue, MS# 11-HLS, Hartford, CT 06134 USA; 7 New York State Department of Health, Bureau of Occupational Health and Injury Prevention, Empire State Plaza, Corning Tower, Room 1325, Albany, NY 12237 USA; 8 Division of Public Health, Nebraska Department of Health and Human Services, Epidemiology and Health Informatics Unit, PO Box 95026, Lincoln, NE 68509 USA; 9 Georgia Department of Public Health, Injury Prevention Program, 2 Peachtree Street NW, 10.414, Atlanta, GA 30303 USA; 10 National Study Center for Trauma and EMS, University of Maryland Baltimore, 110 South Paca Street, Baltimore, MD 21201 USA

**Keywords:** Motorcycle helmet, Motorcycle helmet law, Legislation, Injury, Probabilistic linkage, Hospital charges, Charges, Traumatic brain injury, Log-binomial regression, Motor vehicle crash

## Abstract

**Background:**

Despite evidence that motorcycle helmets reduce morbidity and mortality, helmet laws and rates of helmet use vary by state in the U.S.

**Methods:**

We pooled data from eleven states: five with universal laws requiring all motorcyclists to wear a helmet, and six with partial laws requiring only a subset of motorcyclists to wear a helmet. Data were combined in the Crash Outcome Data Evaluation System’s General Use Model and included motorcycle crash records probabilistically linked to emergency department and inpatient discharges for years 2005-2008. Medical outcomes were compared between partial and universal helmet law settings. We estimated adjusted relative risks (RR) and 95 % confidence intervals (CIs) for head, facial, traumatic brain, and moderate to severe head/facial injuries associated with helmet use within each helmet law setting using generalized log-binomial regression.

**Results:**

Reported helmet use was higher in universal law states (88 % vs. 42 %). Median charges, adjusted for inflation and differences in state-incomes, were higher in partial law states (emergency department $1987 vs. $1443; inpatient $31,506 vs. $25,949). Injuries to the head and face, including traumatic brain injuries, were more common in partial law states. Effectiveness estimates of helmet use were higher in partial law states (adjusted-RR (CI) of head injury: 2.1 (1.9-2.2) partial law single vehicle; 1.4 (1.2, 1.6) universal law single vehicle; 1.8 (1.6-2.0) partial law multi-vehicle; 1.2 (1.1-1.4) universal law multi-vehicle).

**Conclusions:**

Medical charges and rates of head, facial, and brain injuries among motorcyclists were lower in universal law states. Helmets were effective in reducing injury in both helmet law settings; lower effectiveness estimates were observed in universal law states.

## Background

Motorcycle helmet laws in the United States vary from state to state, and range from no law requiring helmets, partial laws covering a portion of motorcyclists usually delineated by age, and universal laws requiring helmet use among all motorcyclists. In 1967 the U.S. government incentivized state legislatures to enact motorcycle helmet laws through highway construction funding. By 1975, 48 of 50 states and Washington DC had enacted universal helmet laws. Despite growing evidence of the effectiveness of motorcycle helmets in reducing head and brain injuries, many state legislatures have repealed or reduced the coverage of those laws since that time [12, 14, 19]. Despite falling rates of motor vehicle crash morbidity and mortality, rates of motorcycle morbidity and mortality have risen since the early 1990’s, peaked in 2007 and 2008, and remained somewhat stable over the last decade [2, 23, 24].

The most recent reductions in state motorcycle helmet laws occurred in Florida (2000), Pennsylvania (2003), and Michigan (2012). Studies have shown increased fatalities and hospitalizations in those states following law reductions [4, 18, 21]. Louisiana re-instated a universal law in 2004 after it was repealed in 1999. An analysis of fatality data showed an increase in fatalities following the repeal. However, the fatality rate following re-enactment did not return to pre-repeal levels [30].

Cross-sectional studies have used fatality data or hospital data from states with differing helmet laws to show a benefit of universal laws compared to partial laws in reducing fatalities, hospitalizations, injuries, and medical charges [5, 13, 20]. However, some of these studies lack patient-level crash details including whether a helmet was used. Others lack medical outcome data including details of non-fatal injuries. Studies that have linked crash details to hospital data have shown a benefit of motorcycle helmets in reducing injuries and fatalities but have not compared the effectiveness of helmets in partial law states to universal law states [6]. It is unclear how estimates of helmet use effectiveness may differ between helmet law settings given the differing rates of helmet use and other state-level factors, including the history of repeal and re-enactment of helmet laws and their influence on motorcyclists’ decisions to use helmets.

In this study we used multi-state linked crash and hospital datasets to compare medical outcomes between motorcyclists who crashed in universal law states to those who crashed in partial law states. We estimated the effectiveness of motorcycle helmets in preventing head, facial, and brain injuries overall, and within each state, in order to compare helmet use effectiveness between helmet law settings.

## Methods

This is a cross-sectional comparison of probabilistically linked motor vehicle crash reports, emergency department records, and hospital billing records from five universal law states and six partial law states.

### CODES Network

Data analyzed in this study were compiled by analysts in eleven states in the Crash Outcome Data Evaluation System (CODES) Network. The CODES network is a collection of states, partially supported by the National Highway Traffic Safety Administration (NHTSA), working together to share probabilistically linked data and support highway safety traffic activities. States contributing data for this analysis were Connecticut, Georgia, Kentucky, Maryland, Minnesota, Missouri, Nebraska, New York, Ohio, South Carolina, and Utah.

### Probabilistic linkage

Trained analysts in each state probabilistically linked police crash records to both hospital emergency department and inpatient discharge records using CODES2000 software (Strategic Matching, Inc. 2000, Morrisonville, New York). Probabilistic linkage is a method that utilizes personal and event information common to a pair of records to estimate the probability that the two records describe the same person and/or event [7, 15]. The type of linkage information available varied from state to state, necessitating linkage models tailored to each state’s datasets and resulting in varying linkage rates. The CODES Technical Resource Center at the University of Utah provided support to ensure the process was as similar as possible for each state. Each state produced five imputed datasets for each year of available data from years 2005-2008. Some states were not able to provide data for all four years. Ten states contributed 2005 data, seven contributed 2006 data, six contributed 2007 data, and eight contributed 2008 data. All available data were included in analyses.

The University of Utah Institutional Review Board approved the use of these data for this study.

### Dataset mapping and multiple imputation

CODES analysts mapped motor vehicle crash record and linked hospital data to a standardized set of data elements and transferred de-identified datasets to the CODES Technical Resource Center for combination and analysis. The process included CODES state analysts creating algorithms for mapping state crash and hospital data to a general used dataset model. These algorithms were reviewed and approved by the Technical Resource Center with assistance from NHTSA’s State Data System. Once approved, linked data were submitted to the Technical Resource Center [8]. Missing data were then imputed using sequences of regression models implemented in IVEware (University of Michigan, Ann Arbor, Michigan) [26, 32]. Due to a high degree of variability of available information between states and years, state and year-specific imputation models were developed. Variables that were not captured by a state’s crash record or hospital databases were not imputed. Rates of imputation varied within and between states. Analysis variables most frequently missing and imputed were: urban/rural location (30 %), and helmet use (14 %). Medical outcomes were rarely missing among linked motorcyclists.

After imputation, data from the eleven states were combined and analyzed. All analyses from multiple imputed data were combined using standard methods [27, 29].

### Analysis data set

The final combined data set consists of motor vehicle crash records for all motorcycle operators involved in state-reported crashes. The dataset additionally contains medical information for motorcycle operators whose crash record linked to an emergency department or inpatient record.

A crash was defined as a night time crash if it occurred between 9:00 pm and 6:00 am. The location of the crash was categorized according to the Federal Highway Administration performance monitoring system definition, which categorizes a population under 5000 as rural. Poor surface conditions included snow, slush, ice, and wet roads. Crash details were used to categorize each crash as a single or multi-vehicle crash, and to determine if the crash was at an intersection. Speed-relatedness was assigned to operators based on contributing factors or similar state crash file attributes. Police suspicion of alcohol or drug use, and police-reported helmet use, were included in the dataset.

Medical outcomes were derived from linked emergency department and inpatient records and include billing information related to the visit such as billed charges, length of stay, and discharge status. International Classification of Diseases, 9th Revision Clinical Modification (ICD-9-CM) codes were used to derive injury severity scores in ICDMAP-90 software (Johns Hopkins University and Tri-Analytics, Baltimore, Maryland). ICD-9-CM codes were also used to derive the body region and nature of each injury using the Barell Injury Diagnosis Matrix [3].

### Study population

This study included 73,759 operators of motorcycles. Operators of parked motorcycles and those involved in crashes occurring outside of the traffic way were excluded. Analyses of medical outcomes were limited to motorcycle crash records with linked emergency department or inpatient records.

### Helmet laws

Of the eleven participating states, universal laws were in force in five states: Georgia, Maryland, Missouri, Nebraska, and New York.

Partial laws were in force in six states: Connecticut, Kentucky, Ohio, Minnesota, South Carolina, and Utah. Helmet use laws varied, with age restrictions for un-helmeted riders ranging from 17 to 20 years-old. In some states, provisions required helmets for those with instructional/learner’s permits and proof of medical insurance for un-helmeted riders. There were no changes to helmet laws in any of the eleven states during the study period [14].

### Statistical analyses

Helmet use rates were compared between motorcycle operators in partial and universal law states. We then compared helmet use rates of motorcycle operators under age 21 in universal law states to those covered by partial laws according to their age in partial law states.

We described medical care received by linked motorcyclists using relative frequencies. Emergency department and inpatient charges were adjusted for yearly inflation to 2008 dollars using the medical care consumer price index, and normalized for differences between state incomes using the population and income estimates from the Bureau of Economic Analysis [33, 34]. Adjusted emergency department and inpatient charges and hospital length of stay were compared using medians and means. We also compared record linkage rates and the rate of fatalities reported at the scene of the crash between helmet law settings.

We described the most-often injured body regions of linked motorcyclists by helmet law and tested for associations using likelihood ratio tests of homogeneity. For linked motorcyclists, we analyzed the rates of three types of injuries that motorcycle helmets are known to effectively reduce the risk of sustaining: (1) head injury, (2) facial injuries, and (3) traumatic brain injury. For motorcyclists who died at the scene or linked to a medical record, we analyzed the rate of moderate to severe head or facial injury or death [6]. Data from the emergency department and inpatient records were combined to identify these injuries. Head injury included traumatic brain injury, other head, or head/face/neck unspecified injuries. Facial injuries included face or head/face/neck unspecified injuries. In addition to linked motorcyclists, analyses of moderate to severe head or facial injury or death also included motorcyclists who died at the scene. This outcome was defined as a head or facial injury with an abbreviated injury severity score of ≥ 2 (moderate), or death by any cause according to either the crash record or the emergency department or inpatient record.

Rates of injury outcomes among linked motorcyclists were estimated for each state separately and by helmet law, with 95 % confidence intervals. We estimated unadjusted relative risks of each injury for linked motorcyclists in partial law states compared to those in universal law states using log-binomial regression models controlling for correlation between motorcyclists within a state [35]. We then examined the effect of helmet use on injury using generalized log-binomial regression models. Relative risk estimates were adjusted for the following: state (as a cluster-effect), gender, age, intersection related, night-time (9:00 pm to 5:59 am), poor surface conditions, year, type of crash (single vs. multi-vehicle), helmet law, and helmet use. We included interactions between helmet use and type of crash, and between helmet use and helmet law. We then fit a model to each state separately in order to estimate within-state relative risks associated with helmet use.

Suspicion of alcohol or drugs, rural/urban location, and speed-relatedness were not available as covariates for all eleven states, and were not included in the primary regression models. In sensitivity analyses, we included these in state-level models when available.

As a sensitivity analysis, we re-estimated unadjusted and adjusted relative risks for all motorcycle operators—including those that did not link to a medical record. This sensitivity analysis made the assumption that non-linked motorcyclists were uninjured.

We used SAS software version 9.3 (SAS Institute Inc. 2002, Cary, North Carolina) for all analyses, and combined results from multiple imputed datasets using the MIANALYZE procedure. We used a 0.05 level of significance for all statistical tests.

## Results

### Description of the study population

This study included 73,759 motorcycle operator crash records, with 28,207 (38 %) records submitted from six partial law states, and 45,552 (62 %) submitted from five universal law states. The 6 partial law states contributed 15 state/years, and the 5 universal law states contributed 16 state/years of data. Crash and operator characteristics are given in Table 1.

**Table 1:** Description of the study population by motorcycle helmet law

Characteristic	Partial helmet law	Universal helmet law	*P*-value*
Number of motorcycle operators	*N* = 28,207	*N* = 45,552	
Helmet used	42 %	88 %	<0.01
Age (Median)	37	36	0.52
Male	93 %	93 %	0.77
Single Vehicle Crash	39 %	45 %	<0.01
Crash at Intersection	36 %	40 %	<0.01
Night time	18 %	17 %	<0.01
Speed related^a^	10 %	17 %	<0.01
Suspicion of alcohol or drugs^a^	9 %	5 %	<0.01
Rural location^a^	17 %	31 %	<0.01
Poor Surface Conditions	6 %	7 %	<0.01
Crash record linked to a medical record	55 %	58 %	<0.01
Died at the scene	3 %	3 %	0.62

^a^ Available for only a subset of the 11 included states: speed related (10), suspicion of alcohol or drugs (9), and rural location (8)

*P-value from a Median regression model with bootstrapped standard error (age), or likelihood ratio test of homogeneity (all others)

Reported helmet use was 42 % in partial law states and 88 % in universal law states. Helmet use rates ranged from 29 % to 54 % in partial law states and from 85 % to 92 % in universal law states. Among those operators covered by an age-related provision in their state’s partial law (*N* = 1660), helmet use was 44 %. In comparison, operators under age 21 in universal law states (*N* = 4166) showed a helmet use rate of 81 %.

Probabilistic linkage methods matched hospital emergency department or inpatient records to 59 % of motorcycle crash records. Linkage rates varied between states, and ranged from 39 % to 65 % in universal law states, and from 50 % to 59 % in partial law states. Among linked motorcyclists, helmet use was similar to overall rates (39 % partial law, 88 % universal law).

Many crash characteristics differed statistically between partial and universal law states including speed relatedness (10 % partial, 17 % universal, *p* < 0.01), alcohol or drug suspicion (9 % partial, 5 % universal, *p* < 0.01), and rural location (17 % partial, 31 % universal, *p* < 0.01). However, location was not available for three partial law states, limiting the usefulness of this comparison.

### Analysis of medical outcomes

Table 2 describes medical outcomes by helmet law for linked motorcyclists. Emergency department and hospital charges were higher in partial law states compared to universal law states. Public/government or self/uninsured payers were responsible for payment more often among motorcyclists in partial law states compared to universal law states. Although hospital length of stay was slightly lower among motorcyclists in partial law states, the percent of patients discharged home was also lower.

**Table 2:** Comparison of medical care received by motorcycle operators involved in crashes in partial and universal helmet law states

	Partial helmet law	Universal helmet law	*P*-value*
Number of motorcycle operators with a linked medical record	*N* = 15,458	*N* = 26,513	
Percent linked to an Emergency department (ED) record	73 %	68 %	
Median ED charges	$1,987	$1,443	<0.01
Mean ED charges	$3,688	$3,217	<0.01
% Public/Self/Uninsured payer	33 %	29 %	<0.01
Percent linked to a Hospital record	27 %	32 %	
Median hospital charges	$31,506	$25,949	<0.01
Mean hospital charges	$59,032	$56,325	0.16
Mean hospital length of stay (days)	6.7	7.1	0.06
Percent discharged home	81 %	83 %	0.03
% Public/Self/Uninsured payer	34 %	29 %	<0.01
Percent died according to ED or hospital record^a^	2.2 %	2.1 %	0.60

*ED* Emergency Department

^a^ One state is excluded due to ED Disposition not being included in the state dataset

* *P*-value from a Median regression model with bootstrapped standard errors (Medians), linear regression model (Means), or likelihood ratio test of homogeneity (Percents)

### Description of injuries

The most often injured body region was the extremities (Table 3). Head and neck injuries were the second most prevalent among emergency department visits, and third behind torso injuries for inpatient admissions. There were significantly less head and neck injuries, including traumatic brain injury, in universal law states compared to partial law states (*p*-values all <0.01). There were more extremity injuries, and slightly fewer other and unspecified injuries in the universal law states compared to partial law states. The majority (80 %) of injuries to other and unspecified body regions were contusions and superficial injuries.

**Table 3:** Body regions injured among motorcycle operators seen in the emergency department or admitted to the hospital

	Emergency department			Hospital		
Body region	Partial	Universal	*P*-value	Partial	Universal	*P*-value
	*N* = 11,261	*N* = 18,057		*N* = 4,197	*N* = 8,456	
Head and Neck	32 %	18 %	<0.01	51 %	41 %	<0.01
Traumatic Brain Injury	8 %	5 %	<0.01	34 %	29 %	<0.01
Spine and Back	10 %	9 %	0.17	18 %	18 %	0.36
Torso	22 %	20 %	0.32	48 %	47 %	0.24
Extremities	72 %	74 %	<0.01	76 %	79 %	<0.01
Other and Unspecified	25 %	23 %	<0.01	11 %	11 %	0.22

Multiple body regions per motorcyclist were included

*P*-values are from likelihood ratio tests of homogeneity comparing universal vs. partial helmet law states

**Fig. 1:**
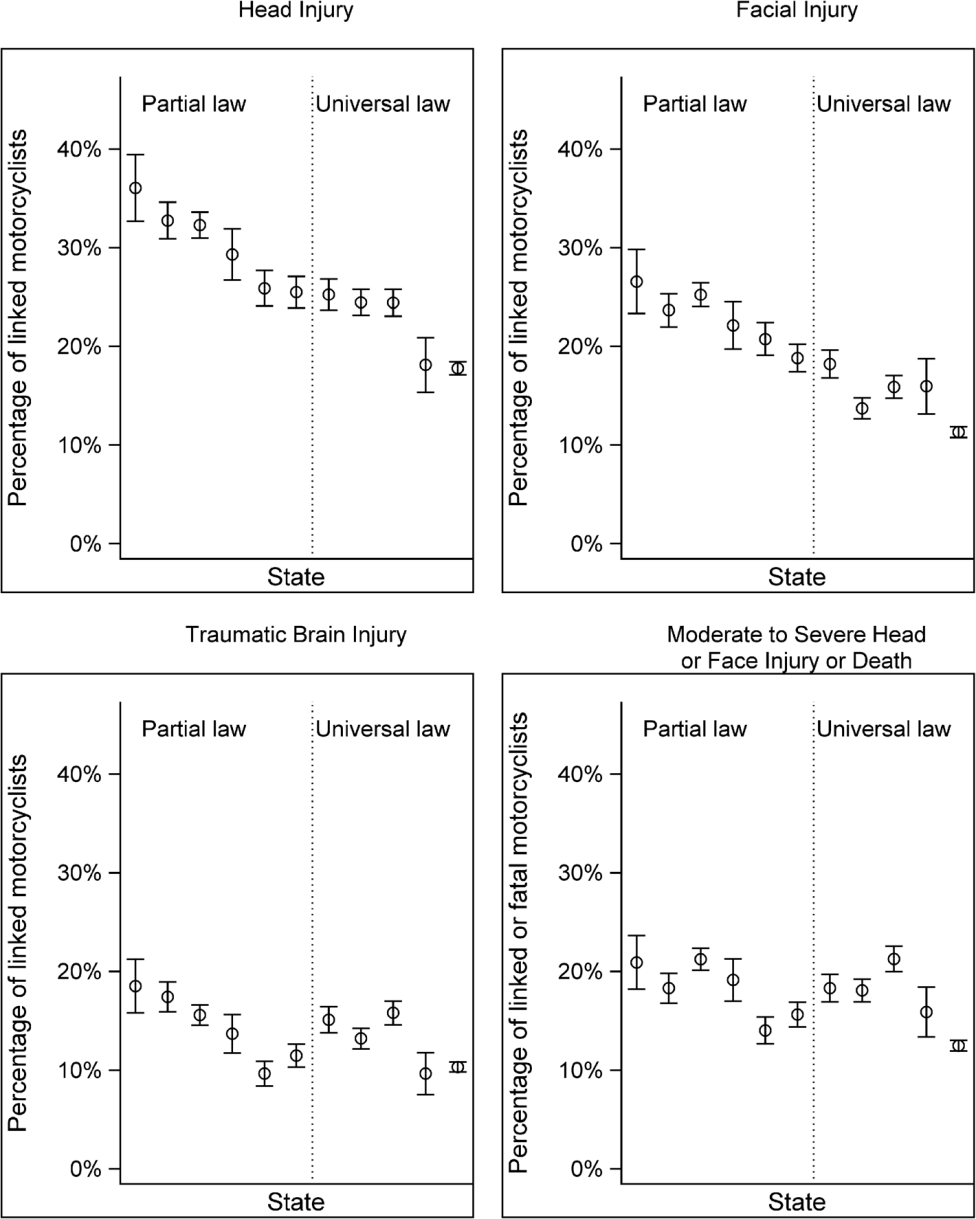
Rates of medical outcomes among motorcyclists in each state in the study. Rates are the percentage of all linked motorcyclists with the injury. In the case of moderate to severe head or face injury or death, rates additionally include motorcyclists who died according to the crash report

Figure 1 shows the rates of head, facial, traumatic brain, and moderate to severe head/facial injuries for the eleven participating states according to emergency department or inpatient records. Table 4 summarizes these, and compares rates in partial law states to those in universal law states.

**Table 4:** Rates and relative risks of death according to the crash report, medical resource utilization, and medical outcomes with 95 % confidence intervals

Motorcycle helmet law	Death according to crash report^c^	Link to medical record^c^	Head injury^a^	Facial injury^a^	Traumatic brain injury^a^	Moderate to severe head or facial injury or death^b^
Partial Helmet Law	3.5 % (3.3,3.7)	55 % (54,55)	30 % (29,31)	23 % (22,23)	14 % (14,15)	18 % (18,19)
Universal Helmet Law	3.4 % (3.2,3.6)	58 % (58,59)	21 % (20,21)	13 % (13,14)	12 % (12,13)	16 % (15,16)
Relative Risk^d^						
Partial vs. Universal Law	1.0 (0.8, 1.2)	1.0 (0.9, 1.2)	1.4 (1.2, 1.6)	1.5 (1.3, 1.8)	1.1 (0.9, 1.4)	1.1 (0.9, 1.3)

^**a**^ Motorcycle operators with linked medical records (ED or hospital) are included

^b^ Motorcycle operators with linked medical records (ED or hospital) and those dead at the scene are included

^c^ All studied motorcycle operators are included

^**d**^ Relative risks control for correlation within state using Generalized Log-Binomial regression models

The rates of head and facial injuries were consistently higher in partial law states compared to universal law states. Facial injuries were 1.5 times more prevalent in partial law states compared to universal law states. Rates of traumatic brain injury differed by 2 percentage points (14 % partial law, 12 % universal law). When traumatic brain injury was further sub-classified in terms of severity, the biggest difference was in the most severe injuries, which were present in 6 % of medically treated patients in partial law states, and 4 % of those in universal law states.

### Modeling the effect of helmet use

We estimated the adjusted relative risk of each outcome (head injury, facial injury, traumatic brain injury, and moderate to severe head or facial injury) separately with multivariable models adjusting for state, year, age, type of crash (single vs. multi-vehicle), intersection, time of day, surface conditions, and helmet law. Interactions between helmet use and type of crash, and between helmet use and the type of law were significant in all four models (p-value for both interactions in all four models <0.01), demonstrating that the effect of helmet use is confounded by the type of crash and the type of helmet law. Conditional adjusted relative risks associated with helmet use are given for the four combinations of helmet law and type of crash (Table 5). In all cases, helmet use was associated with a reduced risk of injury. Not wearing a helmet was associated with an increased risk of facial injuries by a factor of 2.7 in single-vehicle crashes in partial law states, and by 1.22 in multi-vehicle crashes in universal law states. Non-helmet use was associated with an increased risk of between 1.2 and 2.1 for head injury, between 1.2 and 1.8 for traumatic brain injury, and between 1.1 and 1.9 for moderate to severe head/face injury or death. Single vehicle crashes in partial law states saw the most benefit of helmet use across the injury types.

**Table 5:** Adjusted relative risks of medical outcomes for no helmet vs. helmet used

Motorcycle helmet law and type of crash	Head injury^a^	Facial injury^a^	Traumatic brain injury^a^	Moderate to severe head or facial injury or death^b^
Partial Law, Single-Vehicle	2.1 (1.9,2.2)	2.7 (2.2,3.4)	1.8 (1.6,2.0)	1.9 (1.6,2.1)
Universal Law, Single-Vehicle	1.4 (1.2,1.6)	1.5 (1.2,1.7)	1.4 (1.2,1.6)	1.4 (1.2,1.6)
Partial Law, Multi-Vehicle	1.8 (1.6,2.0)	2.3 (1.8,2.9)	1.5 (1.3,1.8)	1.5 (1.3,1.7)
Universal Law, Multi-Vehicle	1.2 (1.1,1.4)	1.2 (1.0,1.4)	1.2 (1.0,1.4)	1.1 (1.0,1.2)

Relative risks are adjusted for state, year, gender, age, intersection, night-time, and poor surface conditions using Generalized Log-Binomial regression models. 95 % Confidence Intervals are shown

^**a**^ Motorcycle operators with linked medical records (ED or hospital) are included

^b^ Motorcycle operators with linked medical records (ED or hospital) and those dead at the scene are included

**Fig. 2:**
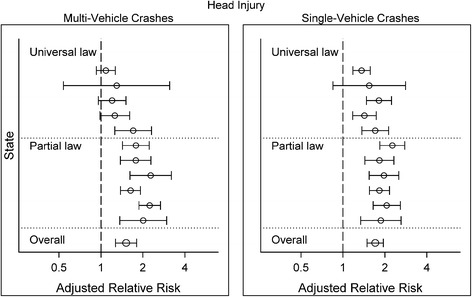
Adjusted relative risks of head injury with 95 % confidence intervals for each state and overall. Estimates for multi- and single-vehicle crashes are shown separately

**Fig. 3:**
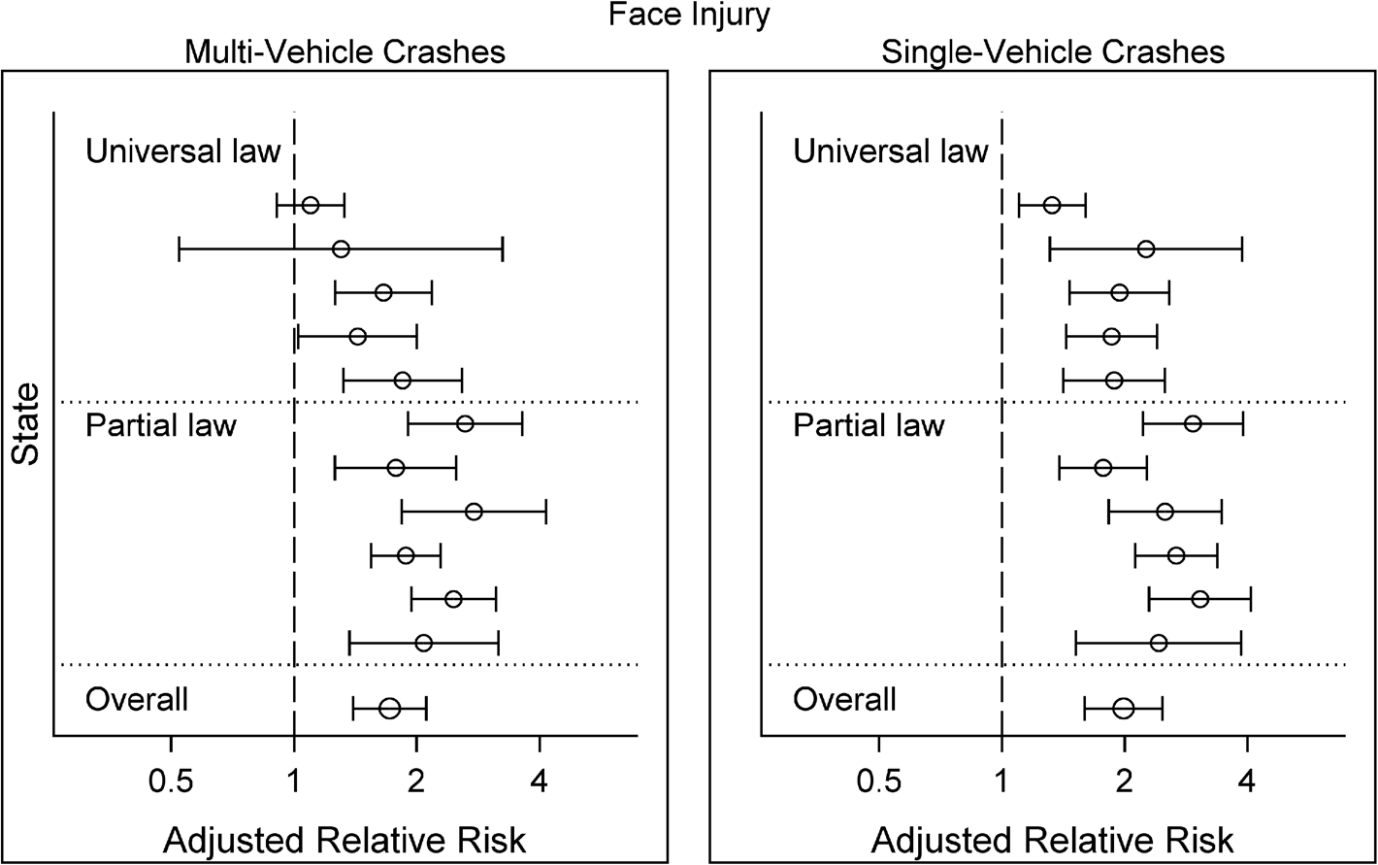
Adjusted relative risks of face injury with 95 % confidence intervals for each state and overall. Estimates for multi- and single-vehicle crashes are shown separately

**Fig. 4:**
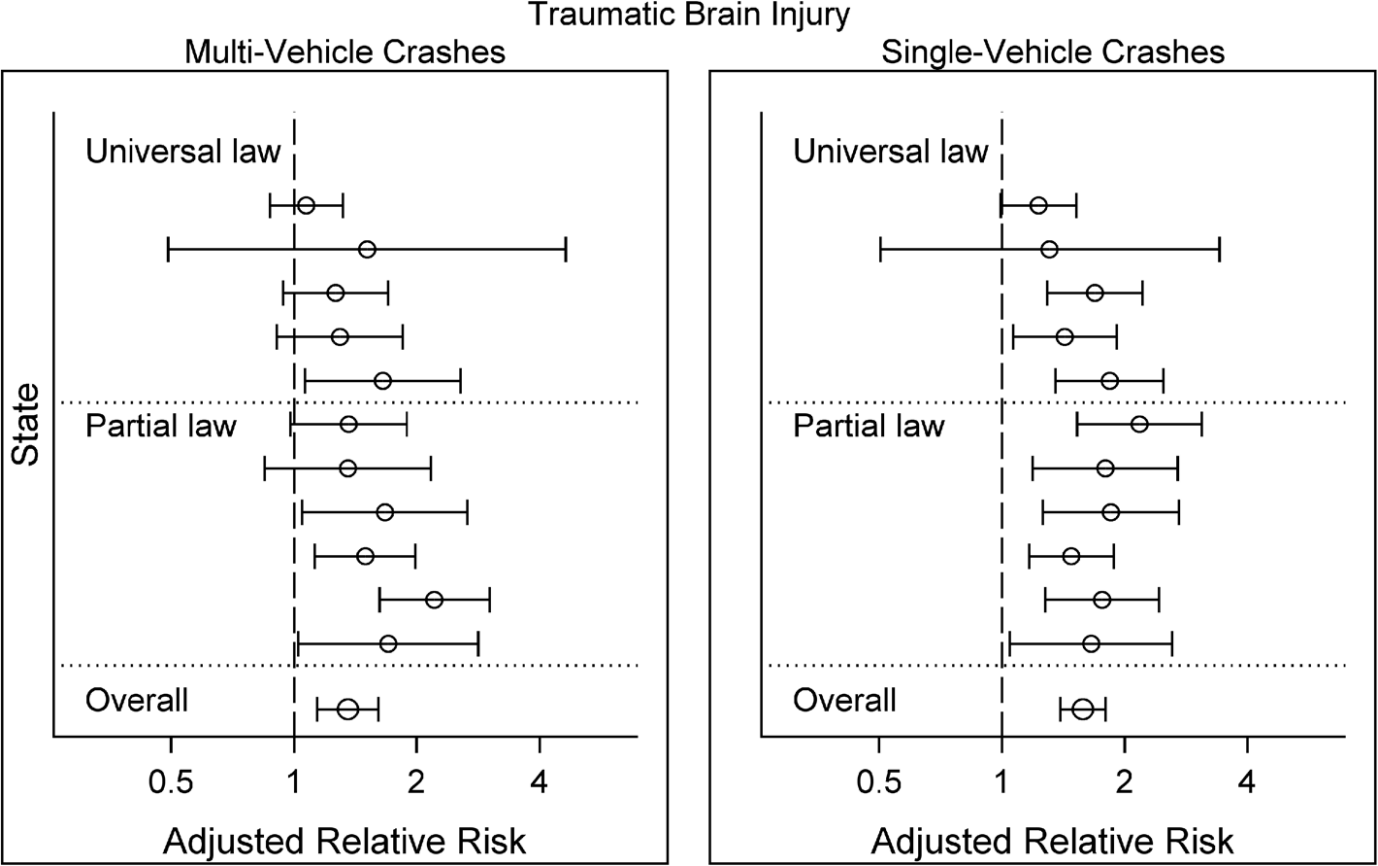
Adjusted relative risks of traumatic brain injury with 95 % confidence intervals for each state and overall. Estimates for multi- and single-vehicle crashes are shown separately

**Fig. 5:**
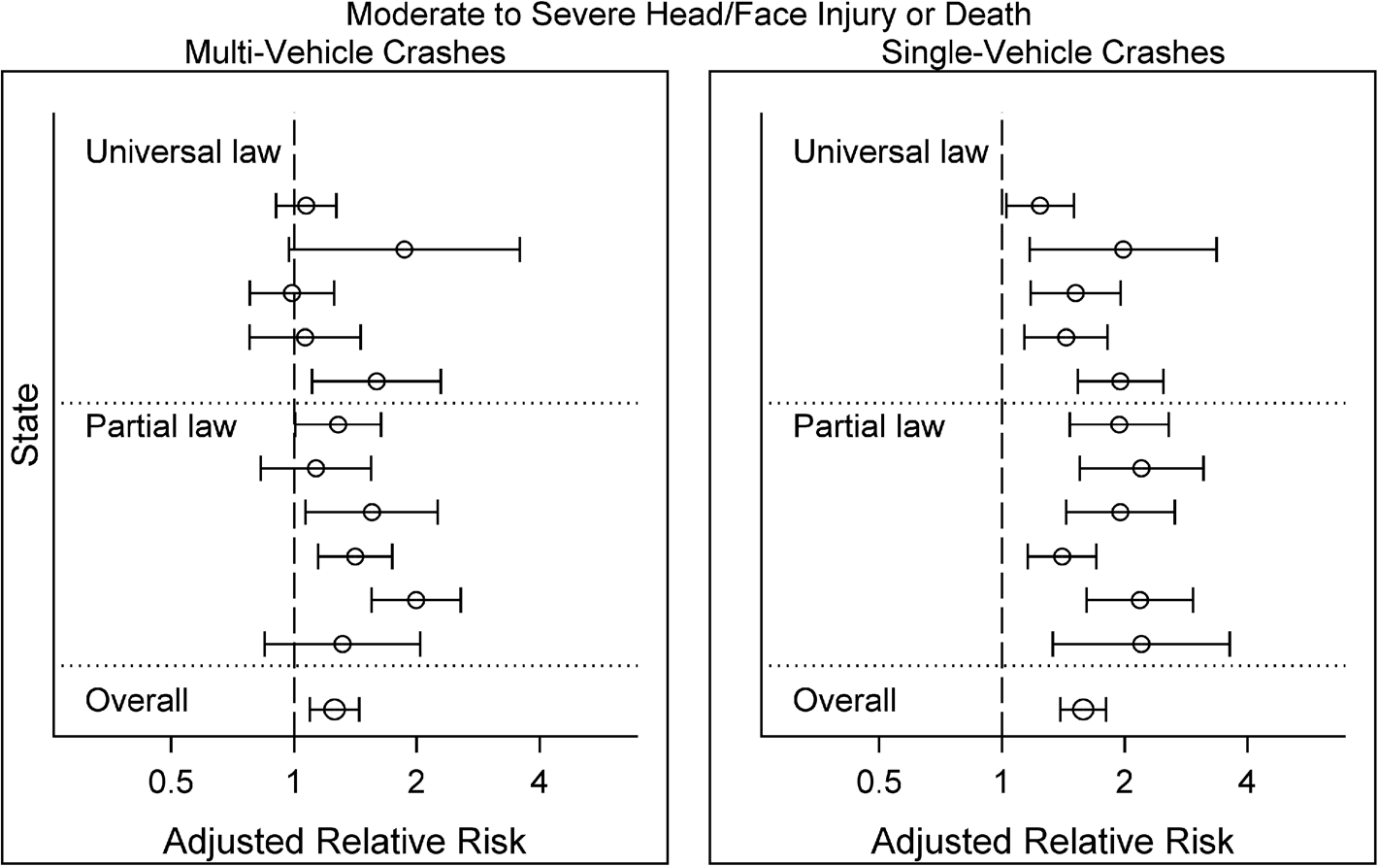
Adjusted relative risks of moderate to severe head or face injury, or death with 95 % confidence intervals for each state and overall. Estimates for multi- and single-vehicle crashes are shown separately

Figures 2, 3, 4 and 5 show the adjusted relative risks of head injury, facial injury, traumatic brain injury, and moderate to severe head/face injury or death for each state, along with the 95 % confidence intervals. Confidence interval widths differed between states due to varying numbers of linked motorcyclists in each state and other state-specific factors. One universal law state had relatively wide confidence intervals due to a high helmet use rate and a relatively small number of motorcyclists. Another universal helmet law state had estimates similar to those seen in partial law states. Relative risk estimates from the other four universal helmet law state models were slightly closer to 1.0 (no difference) as a group than those from partial law state models as a group. This dampened effect of helmet use was also seen in the overall model (Table 5), where universal law effect estimates were lower than corresponding partial law estimates.

In sensitivity analyses that included all motorcycle operators and assumed non-linked operators were not injured, unadjusted and adjusted relative risks of injury were very similar to those estimated using linked operators only. Unadjusted relative risks of head, facial, traumatic brain, and moderate to severe head/face injury or death changed by less than 0.1. Adjusted relative risks were either unchanged, or increased by up to 0.3, suggesting a conservative bias, if any, in the reported estimates.

## Discussion

The morbidity and mortality of motorcycle crashes continues to be a public health problem. Using data from a collaboration of eleven states representing universal and partial helmet laws, this study describes the helmet use and medical outcomes of a large number of motorcyclists involved in crashes over a four year period. Motorcycle helmets were associated with reduced risk of head, facial, and traumatic brain injury and death in both universal and partial law settings. However, that effect was less pronounced within universal law states compared to partial law states. Medical outcomes, including emergency department and inpatient charges, were more severe among injured motorcyclists in partial law states. Motorcyclists in partial law states were more likely to sustain head, facial, and traumatic brain injuries compared to those in universal law states.

An enormous amount of research has shown and quantified the effectiveness of motorcycle helmets in reducing morbidity and mortality. Evidence that helmets protect motorcyclists from head injuries and fatalities was published more than 70 years ago [1]. A recent Cochrane review of 61 observational studies estimated that helmet use reduces the odds of death by 42 % and the odds of head injury by 69 % [19]. As states in the U.S. have enacted, repealed, and re-enacted universal helmet laws over the years, many studies have been done to quantify the results of those changes. Systematic reviews of such studies concluded that rates of helmet use were lower, and morbidity and mortality higher, in partial law states compared to universal law states [10, 11]. Reviews of economic studies concluded that universal helmet laws were associated with estimated benefit of between $1.8 million and $27.2 million per 100,000 registered motorcycles per year, and that public programs paid for much of the associated medical costs [10, 11, 17]. Our study corroborates those findings. Helmet use ranged from 85 to 92 percent in universal law states compared to 29 to 54 percent in partial law states. Motorcyclists who were required to wear a helmet according to their age in partial law states wore them only 44 % of the time. Rates of head injury in partial law states were 1.4 times rates in universal law states. Helmet use was associated with a 52 % reduction in the risk of head injury in partial law states (RR for no-helmet vs. helmet in a single-vehicle crash: 2.1, 95 % CI: 1.9-2.2). Emergency department and hospital inpatient charges were lower in universal law states, and the proportion of injured motorcyclists with public insurance was lower in universal law states, compared to partial law states.

Much of the research in this field has utilized crash data such as federal or state fatality or motor vehicle crash databases which lack medical outcomes, or hospital data such as trauma registries, administrative hospital databases, or samples of hospital patients which lack a description of helmet use and other crash characteristics. This study used methods to link state motor vehicle crash records with emergency department and inpatient discharge records, resulting in a uniquely rich dataset with person-level medical outcomes and crash details, including helmet usage. Despite the fact that motorcycle helmets should have the same effect in any state, this dataset showed that helmet use was associated with a lower risk of head, facial, and traumatic brain injuries in partial law states compared to universal law states. In a model including all eleven states, the interaction between helmet law and helmet use was significant (*p* < 0.01) for all four injury types modeled, suggesting confounding effects related to state helmet laws.

Analyzing states individually offered a unique opportunity to study the variability in helmet effectiveness estimates across states and helmet law settings. In state-specific models, helmet use appeared more effective in single-vehicle settings, and more often statistically significant in reducing injuries that were more prevalent (head and facial injuries), which is expected because statistical power increases with more common outcomes. The tendency for helmet use effectiveness estimates to be less dramatic within universal law states compared to partial law states was apparent in these state-specific models.

There are many possible confounding factors which may explain the plateauing effect of helmet use among motorcyclists in universal law states. Confounding factors may include the legal ramifications of using a helmet, the prevalence of non-compliant helmets, and the characteristics of motorcyclists who choose to use or not use a motorcycle helmet. Additionally, there may be confounding factors related to characteristics that differ by location such as weather, traffic congestion, and other motor vehicle laws.

The plateauing effect of helmet use may partially be a result of the threat of legal ramifications. Motorcycle crashes involving injured, un-helmeted motorcyclists may go unreported more often, and un-helmeted motorcyclists with injuries may be more likely to falsely report helmet use in universal helmet law states, because motorcyclists fear legal ramifications. This would lead to a more-severely injured control (non-helmet) group in those states, and a smaller effect.

Motorcyclists in universal law settings may be more likely to wear non-compliant helmets (i.e. novelty helmets, non-FMVSS 218 compliant) in order to avoid a ticket. Many state helmet laws require that motorcycle helmets comply with Federal Motor Vehicle Safety Standard 218, and all motorcycle helmets sold in the U.S. are required to meet this standard. However, many non-compliant helmets are sold as “novelty” helmets, and laws restricting their use are difficult to enforce [25]. The National Occupant Protection Use Survey and studies in California, Maryland, and New York show a non-trivial proportion of motorcyclists using non-compliant helmets [9, 16, 22, 28]. Non-compliant helmets are less effective, if effective at all, in preventing injury than compliant helmets [9, 16]. Non-compliant helmets used in universal helmet law states may be negatively biasing the benefit of those laws and deflating the effectiveness estimates of motorcycle helmet use.

A motorcyclist’s choice to use a helmet is related to many factors which may confound the relationship between helmet use and injury. These choices include, but are not limited to the state helmet law. A motorcyclist who chooses to wear a helmet when not obligated to by law may be more cautious, experienced, or educated than a motorcyclist who chooses not to wear a helmet in the same setting. On the other hand, a motorcyclist may take more risks because he/she feels safer while wearing a helmet. A study by Teoh and Campbell [31] showed associations between riders of sport motorcycles and both helmet use and risky driving behaviors. That study also showed that riders of cruiser and touring motorcycles involved in fatal crashes were less likely to wear helmets and more likely to be involved in alcohol-related crashes. Understanding and quantifying the confounding effects of motorcyclist characteristics is crucial in order to interpret the differences in helmet use effect estimates in the two helmet law settings studied.

This study has limitations. These data are not the most recent data available at the state-level, and may not be representative of the U.S. or other parts of the world. However, helmet laws in these states have not changed since the data were collected; this dataset represents a one-time collaboration; and our results are consistent with those observed in nationally-representative samples. Crash data are limited by the scope and nature of data collection and may not be reliable. For example, alcohol and drug use are not confirmed with blood-alcohol testing, and helmet use is police-reported. Additionally, thresholds for reporting a crash differed by state. However, an injured motorcyclist would meet the threshold in all states, resulting in little impact on this analysis. States did not uniformly collect the type of helmet that was used. More research is warranted to quantify the prevalence and effect of non-compliant helmets in both partial and universal law states. We did not know injured body-regions for non-linked motorcyclists, and therefore limited our main analyses to those with emergency department or inpatient records. Any bias caused by excluding non-linked motorcyclists would result in conservative estimates of the effect of motorcycle helmets or motorcycle helmet laws.

## Conclusions

Motorcycle helmets were associated with reduced risk of head, facial, and traumatic brain injury and death in both universal and partial helmet law settings. This effect was less pronounced in universal law states compared to partial law states. Future research is warranted to identify and quantify confounding factors related to helmet use, helmet laws, and injuries in order to promote safety among motorcyclists in all helmet law settings. Medical outcomes, including emergency department and inpatient hospital charges were higher among motorcyclists in partial law states. Motorcyclists in partial law states were more likely to sustain head, facial, and traumatic brain injuries. These differences support the effectiveness of universal helmet laws.

## Abbreviations

CDC: Centers for Disease Control and Prevention
CI: Confidence interval
CODES: Crash Outcome Data Evaluation System
ICD-9-CM: International Classification of Diseases, 9th Revision Clinical Modification
NCSA: National Center for Statistics and Analysis
NHTSA: National Highway Traffic Safety Administration
